# Supplementary material for: Evidence for Coexistence of Bulk Superconductivity and Itinerant Antiferromagnetism in the Heavy Fermion System CeCo(In1−xCdx)5
Source: Sci Rep. 2015 Jul 30;5:12528. doi: 10.1038/srep12528 (PMC4519736; doi:10.1038/srep12528)
Supplement: Supplementary Information [file srep12528-s1.pdf]

Supplementary information

Evidence for Coexistence of Bulk Superconductivity and Itinerant Antiferromagnetism in the Heavy Fermion System CeCo($In_{1-x}$$Cd_x$)$_5$

Ludovic Howald[1,2,⋆], Evelyn Stilp[1,3], Pierre Dalmas de Réotier[4], Alain Yaouanc[4], Stéphane Raymond[4], Cinthia Piamonteze[2], Gérard Lapertot[4], Christopher Baines[3] & Hugo Keller[1]

[1]*Physik-Institut der Universität Zürich, Winterthurerstrasse 190, CH-8057 Zürich, Switzerland*
[2]*Swiss Light Source, Paul Scherrer Institut, CH-5232 Villigen PSI, Switzerland*
[3]*Laboratory for Muon Spin Spectroscopy, Paul Scherrer Institut, CH-5232 Villigen PSI, Switzerland*
[4]*Université Grenoble Alpes, INAC-SPSMS, F-38000 Grenoble, France and CEA, INAC-SPSMS, F-38000 Grenoble, France*
⋆*ludovic.howald@psi.ch*

S.1 Sample characterization

Single crystals of the tetragonal system CeCo($In_{1-x}Cd_x$)$_5$ were grown by the self–flux technique with different nominal cadmium concentrations (x)[1,2]. The actual cadmium concentration is much less[3] than the nominal concentration x and depends on details of the sample growth. X-Ray Diffraction (XRD) indicates an actual concentration ≈ 10 times smaller than the nominal one. Two types of transitions are observed in the temperature dependence of the specific heat (Fig. S.1). The sharp jump is characteristic of the SC transition (T_c), while the broader transition corresponds to the magnetic order (T_N). The green line in Fig. S.1 represents a fit discriminating the two transitions in sample $x = 0.06$, using the shape of closely related samples ($x = 0.03$ and $x = 0.09$, dashed lines). Following the work of Ref. 4 the chemical pressure produced by the cadmium dopant in CeCo($In_{1-x}Cd_x$)$_5$ samples can be assimilated to a negative hydrostatic pressure (p) on the parent system $CeCoIn_5$. Indeed, under hydrostatic pressure CeCo($In_{1-x}Cd_x$)$_5$ samples have a phase diagram very similar to $CeCoIn_5$ with a shift in the pressure scale of $\simeq 0.14$ GPa per percent of nominal doping x[4,5]. To allow comparison with previous work the samples are named according to their corresponding hydrostatic negative pressures in this work. The corresponding hydrostatic pressure is determined by comparison of the AFM and SC transition temperatures to the ones reported in the literature. Transition temperatures and equivalent pressure for the samples presented here, are reported in table S.1.

S.2 Analysis of the μSR spectra at 1.6 K

The μSR spectra in absence of a magnetic field were fitted using Eq. (3) and a Bessel depolariza-

tion function. The results for the two different initial muon spin orientations ($\|$ and $\perp$) at $1.6\,\mathrm{K}$ are presented in table S.2. The reduced stopping fraction in the sample $p = -0.7\,\mathrm{GPa}$ with configuration $\perp$ compared to the other sample is understood due to a sample holder with larger mount. The background contribution is represented by the dashed line in Fig. 3. The parameters for the temperature dependence are presented in the next section.

S.3 Temperature dependence of the μSR spectra

For the temperature dependence of the ZF μSR spectra, only the configuration $\vec{S} \parallel \vec{c}$ was used.

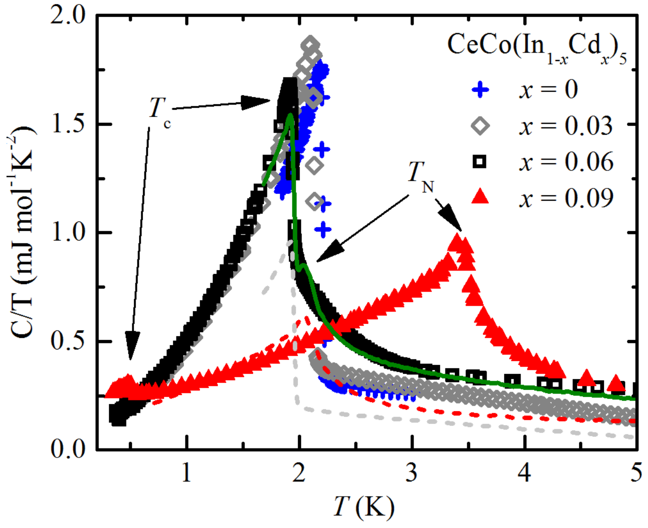

Figure S.1: Temperature dependence of the specific heat in $CeCo(In_{1-x}Cd_x)_5$, x stands for the nominal doping value. The step like transition indicates the onset of superconductivity (T_c), while the broader transition corresponds to the magnetic transition at the Néel temperature (T_N). The green line is a sum of the parameterized curved ($x = 0.03$ and $x = 0.09$, dashed curves), scaled and shifted in temperature to fit the specific heat curve of the $CeCo(In_{1-x}Cd_x)_5$ sample with $x = 0.09$. It was used to determine T_c and T_N for the sample of nominal doping $x = 0.09$, as the features of the two transition temperatures overlap.

x	T_c (K)	T_N (K)	p (GPa)	μSR	XAS
0	2.21(2)	0	0	Ref. 6	Yes
0.03	2.14(2)	< 0.3	-0.4	–	–
0.06	1.95(2)	2.06(2)	-0.7	Yes	–
0.09	0.515(15)	3.44(2)	-1.45	Yes	Yes

Table S.1: SC and AFM transition temperatures for samples of various $CeCo(In_{1-x}Cd_x)_5$ nominal compositions. Corresponding negative hydrostatic pressures and experiments performed are also indicated.

The μSR asymmetry spectra were fitted globally with a simplified version of Eq. (3) ($\alpha = 90°$). This simplification leads to a slight decrease in the fit quality. The perpendicular contribution is in such a model included in the background contribution: Δ_{Bg} was a free, temperature independent parameter. The AFM transition of the two CeCo(In$_{1-x}$Cd$_x$)$_5$ samples ($p = -0.7$ GPa and $p = -1.45$ GPa) can be obtained following the temperature dependence of B_m (Fig. S.2a).

A temperature dependence of the form: $B(T) = B(0)\sqrt{1-(T/T_{\rm N})^{\alpha}}$ was used. The values $\alpha = 1.8(3)$ gives the best agreement for sample $p = -0.7$ GPa. For the sample $p = -1.45$ GPa we found $\alpha = 3.7(7)$ and $T_{\rm N} = 3.38(2)$ K in agreement with the specific heat transition (3.44(2) K). For the sample $p = -0.7$ GPa the transition is more difficult to pinpoint as we observed a phase coexistence in the temperature range 1.6 K< T <2.3 K. In this range, the μSR asymmetry spectra (Fig. S.2b) is the sum of a Kubo-Toyabe and an exponential decay contributions (Eq. (3) with $0 < f_M < 1$). The non-magnetic contribution observed at 1.91 K & 2.06 K is highlighted in Fig. S.2b by the dashed area, that corresponds to a fraction of the normal state contribution (2.27 K). In Fig. S.2b, even a small non-magnetic fraction is clearly observable, as the Kubo-Toyabe function drastically differs from the Bessel depolarization function characteristic of the magnetic phase. In contrary, there is no signs of a Kubo-Toyabe contribution in Fig. 3, indicating that the magnetic fraction is of 100 % below 1.6 K in sample $p = -0.7$ GPa, and in the full temperature range investigated for sample $p = -1.45$ GPa.

The fact that the temperature dependence of the internal field measure by μSR differs from the temperature evolution of the amplitude of the magnetic moment measured by neutron diffraction, might be due to a larger doping distribution in the μSR experiment than in the neutron diffraction experiment. At large length scale such as probed by neutron diffraction, the average antiferromagnetic moment is reduced by the occurrence of superconductivity [7]. This effect should differ on a length scale smaller than the superconducting coherence length such as probed by μSR and is possibly another reason for the different temperature dependencies.

The transition is better observed in the temperature dependence of the damping parameter (λ) measured in longitudinal field ($\mu_0 H = 10$ mT). The μSR asymmetry spectra were fitted using a dynamic Kubo–Toyabe depolarization function. The temperature dependence of λ is presented in

p (GPa)	B_m (mT)	θ (°)	f_M (%)	Δ_P (mT)	Δ_L (mT)	$f_{S\parallel}$ (%)	$f_{S\perp}$ (%)
-0.7	6.5(2)	50(2)	93(1)	2.3(1)	1.41(1)	86(1)	27(1)
-1.45	11.2(1)	74(2)	100(1)	3.1(1)	4.0(7)	75(1)	51(3)

Table S.2: Parameters for the fit of the μSR asymmetry of the CeCo(In$_{1-x}$Cd$_x$)$_5$ sample at 1.6 K (Fig. 3). B_m is the amplitude of the maximum magnetic field at the muon stopping site, θ the angle between $\vec{B_\mu}$ and the sample $\vec{c}$-axis, f_M the magnetic fraction and $f_{S(\perp/\parallel)}$ the fraction of muons stopping in the sample, for the two experimental configurations. Δ_P and Δ_L are the two damping rates defined in Eq. (3).

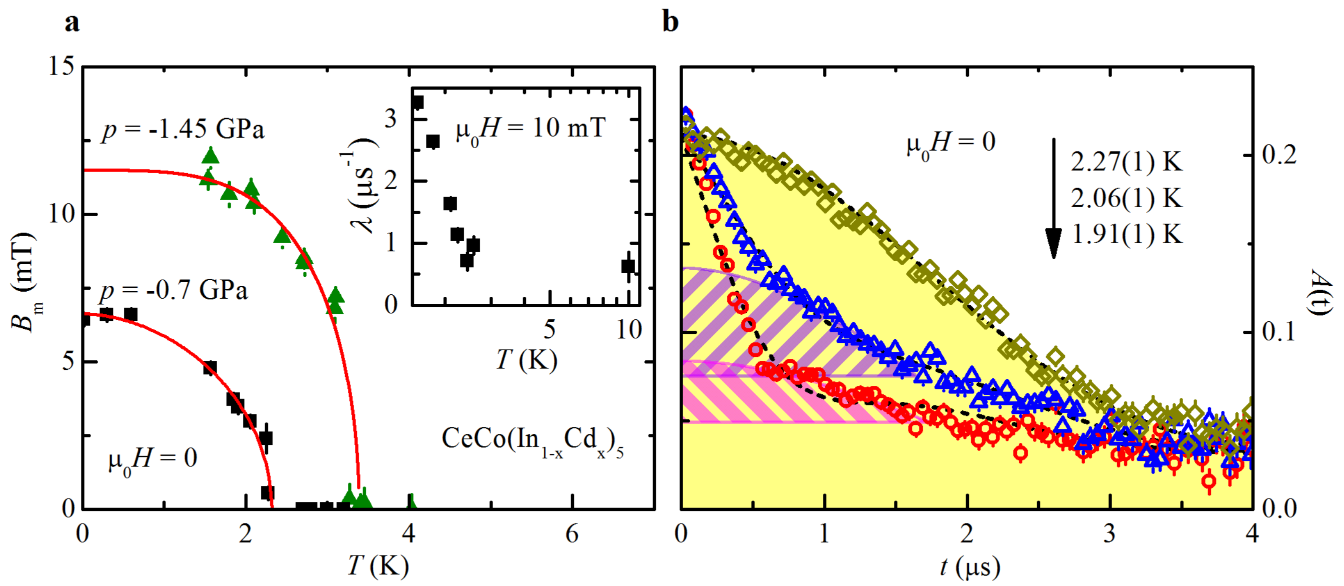

Figure S.2: (**a**) Temperature dependence of the internal magnetic field for $CeCo(In_{1-x}Cd_x)_5$ $p = -1.45\,$GPa and $p = -0.7\,$GPa. In the sample $p = -0.7\,$GPa a phase separation between an antiferromagnetic and non-magnetic (represented by the dashed areas) part is observed in the range 1.6 K< T <2.3 K (**b**). For the same sample the temperature dependence of the relaxation rate λ in a longitudinal field is reported in the inset of panel (**a**).

inset of Fig. S.2a. It gives $T_{\mathrm{N}} \simeq 2.31$ K, which is substantially higher than the specific heat transition (2.06(2) K), suggesting some distribution in the cadmium dopant concentration. From the phase diagram (Fig. 1), this doping distribution can be estimated to a maximum of $\delta p < 0.15\,GPa$ that corresponds to an effective doping variation of $\delta x < 0.001$. Such a small doping distribution can occur between the different single crystals forming the sample mosaic or even within a single crystal.

S.4 Valence of reference systems for XAS measurements

The valences of the reference systems CeF_3 and CeO_2 were extracted via the temperature dependence of the susceptibility of the two systems. Indeed, CeF_3 and CeO_2 do not have necessarily pure 3+ and 4+ valences due to the possible formation of CeF_4 and Ce_2O_3 phases. The effective valences were measured via the moment of the cerium extracted from the Curie temperature dependence of the susceptibility: $\chi(T) = C/T$. The Curie constant has the form:

$$C = \frac{\mu_0 \mu_B^2}{3k_B} N_A g_J J(J+1) \quad (5)$$

where μ_0 is the vacuum permeability, μ_B is the Bohr magneton, k_B is the Boltzmann constant, N_A is the Avogadro number, g_J is the Landé g-factor, and J is the angular momentum. The magnetic moment is obtained as:

$$m = g_J \sqrt{J(J+1)} \mu_B \quad (6)$$

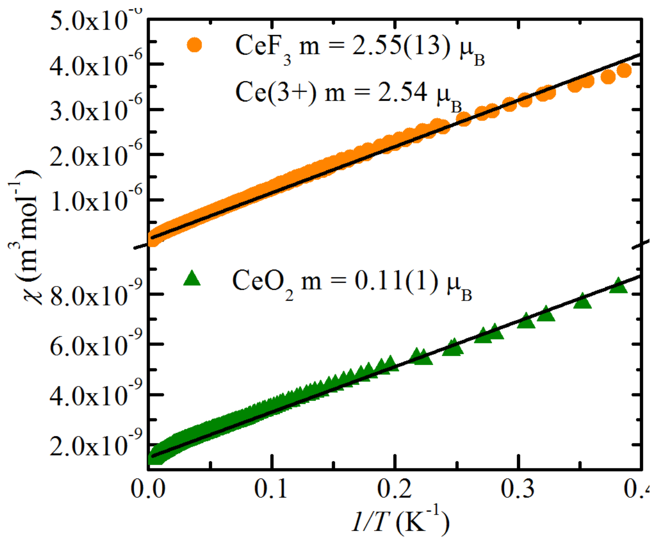

Figure S.3: Low temperatures Curie–Weiss susceptibility of the reference powder insulator systems CeF_3 and CeO_2.

System	ν		dE (meV)		"delocalization"
γ-Ce ($T = 300$ K)	0.398(2)	$\uparrow$	175(2)	$\uparrow$	$\uparrow$
α-Ce ($T \simeq 4$ K)	0.374(1)		158(2)		
$CeCoIn_5$ $p = 0$ GPa	0.142(1)	$\downarrow$	153(1)	$\uparrow$	$\uparrow$
$CeCoIn_5$ $p = -1.45$ GPa	0.154(2)		145(2)		

Table S.3: Evolution of the Ce 4f valence (3+ν) and of the Ce 4f level hybridization (dE), obtained using Eq. (4). The values and errors are given assuming a fixed continuum contribution for each spectrum. The arrows indicate the observed physical "delocalization" (last column), as well as the one expected from the evolution of the parameters ν and dE.

No moment is expected for cerium in the 4+ valence case $m_{4+} = 0$ while for the 3+ contribution, Eq. (6) gives: $m_{3+} = 2.54\,\mu_B$. The susceptibility of the two reference powders was measured in a SC quantum interference device (SQUID) (Fig. S.3). We obtained that the reference systems CeF_3 and CeO_2 are at 100(1) %, respectively 4(1) % in the 3+ state. Including the deviation of CeO_2 from a pure 4+ state has no influences on the results discussed in the main article.

S.5 Parameters for the fits of the XAS spectra and evolution of the XAS spectra of $CeCoIn_5$ under magnetic field

In order to obtain the valence of α and γ metallic cerium as well as of $CeCo(In_{1-x}Cd_x)_5$ $p = 0$ GPa and $p = -1.45$ GPa, their XAS spectra were fitted with Eq. (4). The results are displayed in table S.3.

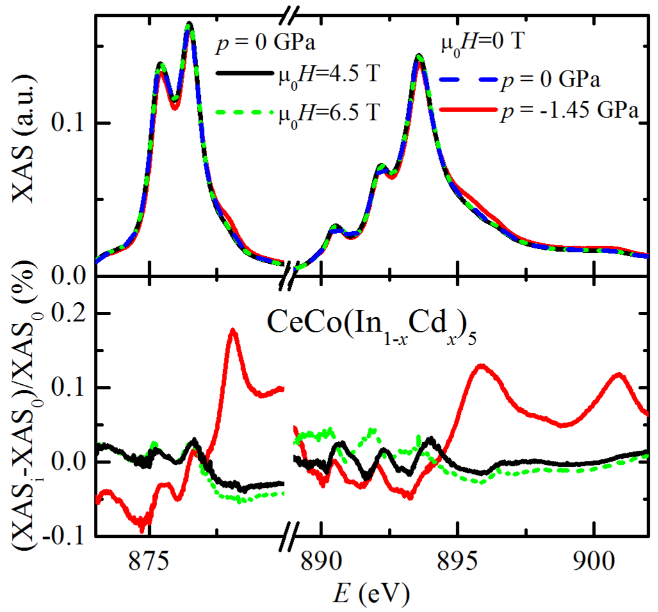

Figure S.4: Evolution of XAS spectra in CeCo(In$_{1-x}$Cd$_x$)$_5$ with doping and under magnetic field. In the bottom panel the normalized difference between the different spectra and a reference one $x = 0$, $H = 0$ at $T \simeq 4\,\mathrm{K}$ is plotted. The regions of low XAS absorption are not presented as the normalized difference between the spectra has less meaning.

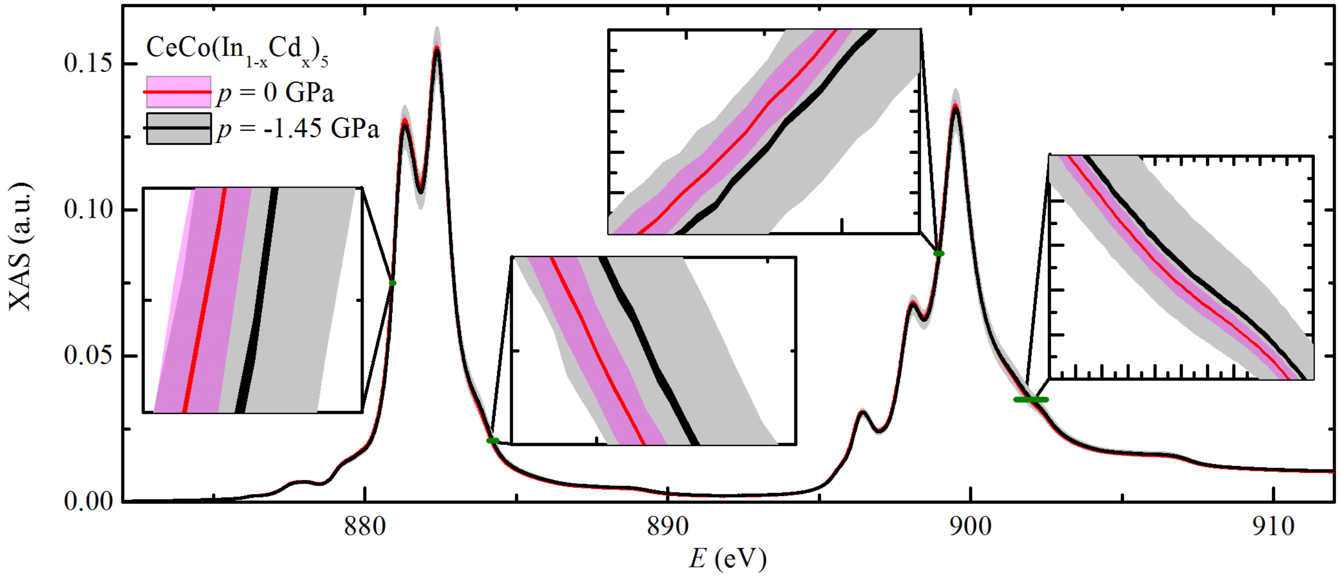

Figure S.5: Resolution of the XAS experiment on $CeCo(In_{1-x}Cd_x)_5$. Insets are details of the XAS spectra at the position indicated by the black and green lines. Full grey and pink areas represent the standard deviation of the spectrum at each energy. Black and red areas represent the error in absolute energy position of the full XAS spectra.

The absence of valence variation in $CeCo(In_{1-x}Cd_x)_5$ with cadmium doping, does not directly imply that the magnetic transition is unrelated to a sizable variation of the valence, as the QCP possibly related to the AFM phase occurs under hydrostatic pressure[8] or magnetic field[9]. However, the magnetic field evolution up to 6.5 T of the XAS spectra of pure $CeCoIn_5$ at low temperature ($\simeq 4\,$K) presented in Fig. S.4 allows such a conclusion. In the lower panel the normalized difference to a reference spectra ($H = 0\,$T $T \simeq 4\,$K) indicates the absence of strong valence variation across the field induced QCP ($H_{QCP} = 4.8\,$T). The differences in XAS spectra between different magnetic fields are in fact smaller than for different doping. The absence of valence variation with cadmium doping in $CeCo(In_{1-x}Cd_x)_5$ and under magnetic field in $CeCoIn_5$ indicates that the “delocalization” mechanism responsible for the AFM transition is not a valence transition.

S.6 Energy stability of XAS spectra

The resolution of the instrument is given by the wavelength bandwidth of the photon beam, which is $\simeq 100\,$meV at the cerium edge. The energy resolution only determines the size of structures in the spectrum which can be resolved. The energy shifts we observe do not have to do with energy resolution, but rather with the energy stability which measures how reproducible or reliable is the photon energy. To quantify the energy stability, we calculated for each spectrum, using the 15 energy scans, the standard deviation at each energy (full grey and pink areas in Fig. S.5). As shown by the different zooms in Fig. S.5 the full curves are systematically shifted one relative to the other. The standard deviation in the position of the full XAS curve is therefore about the

standard deviation of a single energy point divided by the square root of the total number of points with a significant energy shift (red and black area in Fig. S.5). The difference between the two full XAS curves is statistically significant.

1. Canfield, P. & Fisk, Z. Growth of single-crystals from metallic fluxes. *Philos. Mag. B* **65**, 1117–1123 (1992).

2. URL `www.mpc.ameslab.gov`. Rare earth obtained form the Materials Preparation Center, Ames Laboratory, US DOE Basic Energy Sciences, Ames, IA, USA. See: www.mpc.ameslab.gov.

3. Tokiwa, Y. *et al.* Anisotropic effect of Cd and Hg doping on the Pauli limited superconductor $CeCoIn_5$. *Phys. Rev. Lett.* **101**, 037001 (2008).

4. Pham, L. D., Park, T., Maquilon, S., Thompson, J. D. & Fisk, Z. Reversible tuning of the heavy-fermion ground state in $CeCoIn_5$. *Phys. Rev. Lett.* **97**, 056404 (2006).

5. Gofryk, K. *et al.* Electronic Tuning and Uniform Superconductivity in $CeCoIn_5$. *Phys. Rev. Lett.* **109**, 186402 (2012).

6. Howald, L. *et al.* Strong pressure dependence of the magnetic penetration depth in single crystals of the heavy-fermion superconductor $\mathrm{CeCoIn_5}$ studied by muon spin rotation. *Phys. Rev. Lett.* **110**, 017005 (2013).

7. Nair, S. *et al.* Magnetism and superconductivity driven by identical $4f$ states in a heavy-fermion metal. *Proc. Natl. Acad. Sci. U.S.A.* **107**, 9537–9540 (2010).

8. Howald, L., Knebel, G., Aoki, D., Lapertot, G. & Brison, J.-P. The upper critical field of $CeCoIn_5$. *New J. Phys.* **13**, 113039 (2011).

9. Howald, L. *et al.* Behavior of the quantum critical point and the Fermi-liquid domain in the heavy fermion superconductor $CeCoIn_5$ studied by resistivity. *J. Phys. Soc. Jpn.* **80**, 024710 (2011).
